# Supplementary material for: Fourteen-Year Temporal Trends in Patients Hospitalized for Mitral Regurgitation: The Increasing Burden of Mitral Valve Prolapse in Men
Source: J Clin Med. 2022 Jun 8;11(12):3289. doi: 10.3390/jcm11123289 (PMC9225648; doi:10.3390/jcm11123289)
Supplement: Supplementary file 1 [file jcm-11-03289-s001.zip › jcm-1693865-supplementary.pdf]

Table S1. Algorithm for identifying the etiology of MR.

**Among patients hospitalized with a principal or related diagnosis of mitral regurgitation (MR): ICD10–codes: I051; I052; I340;I341**			
**Comorbidities identified by recording the principal, related or associated diagnoses during a previous hospitali stay (2006-2020) and the index stay**	**ICD–10 codes**	**Classification**	
Mitral prolapse or chordae tendineae rupture	I341–I511	Mitral valve prolapse	Primary MR
Rheumatic heart disease	I051–I052	Rheumatic MR	
Ischemic heart diseases	I20–I25	MR with chronic ischemic heart disease	Secondary MR
Another cardiomyopathy code	I42; I43; I517; I518; I519; I52	MR with cardiomyopathy	
Heart failure od who underwent cardiac resynchronization therapy with a triple-chamber cardioverter-defibrillator or pacemaker procdure	I50; I110; I130; I132; codes acts	Other secondary MR	
None	None of those	Unclassified MR	Unclassified MR

Table S2. Characteristics of patients hospitalized for mitral regurgitation, France, 2019.

	**ALL MR**	**Primary MR**	**Secondary MR**	**Unclassified MR**
**Total number, n (% *row*)**	**7232 (100.0%)**	4277 (59.1%)	2127 (29.4%)	828 (11.4%)
**Incidence (per 100,00PY)**				
Crude incidence	10.81	6.40	3.18	1.24
Age standardized incidence	10.78	6.38	3.15	1.25
**Demographic characteristics**				
Age (years), mean (SD)	68.5 (13.9)	67,4 (14,0)	72,1 (12,3)	65.3 (15.1)
**Sex, n (%)**				
Men	4209 (58.2%)	2559 (59.8%)	1207 (56.7%)	443 (53.5%)
Women	3023 (41.8%)	1718 (40.2%)	920 (43.3%)	385 (46.5%)
**Fdep *, n (%)**				
Quintile 1 (the least disadvantaged)	1358 (19.5%)	832 (20.2%)	358 (17.5%)	168 (21.2%)
Quintile 2	1403 (20.2%)	827 (20.1%)	406 (19.9%)	170 (21.4%)
Quintile 3	1477 (21.2%)	897 (21.8%)	431 (21.1%)	149 (18.8%)
Quintile 4	1396 (20.1%)	807 (19.6%)	421 (20.6%)	168 (21.2%)
Quintile 5 (the most disadvantaged)	1324 (19.0%)	760 (18.4%)	425 (20.8%)	139 (17.5%)
**Etiology, n (%)**				
Primary	4277 (59.1%)	4277 (100%)	-	-
Prolapse	3560 (49.2%)	3560 (83.2%)		
Rheumatic	717 (9.9%)	717 (16.8%)		
Secondary	2127 (29.4%)	-	2127 (100%)	-
Chronic ischemic heart disease	1,235 (17.1%)		1,235 (58.1%)	
Cardiomyopathy	319 (4.4%)		319 (15.0%)	
Other secondary MR	573 (7.9%)		573 (26.9%)	
Unclassified	828 (11.4%)	-	-	828 (100.0%)
**Medical characteristics, n (%)**				
Charlson comorbidity index mean (SD)	2.13 (2.14)	1.96 (2.11)	3.1 (2.0)	0.61 (1.42)
History of hospitalization for heart failure, n (%)	2818 (39.0%)	1447 (33.8%)	1371 (64.5%)	0
Mitral valve replacement before 2017, n (%)	123 (1.7%)	57 (1.3%)	61 (2.9%)	5 (0.6%)
Length (days) of index stay mean (SD)	9.1 (11.0)	9.1 (10.8)	10.2 (12.4)	6.3 (7.3)

**Management of mitral regurgitation in the year following the index hospital stay **, n (%)**				
Surgical valve replacement	1835 (25.4%)	1073 (25.1%)	600 (28.2%)	162 (19.6%)
Surgical valve plasty	2805 (38.8%)	2032 (47.5%)	405 (19.0%)	368 (44.4%)
Percutaneous mitral valve repair	933 (12.9%)	555 (13.0)	347 (16.3%)	31 (3.7%)
No mitral procedure mentioned	1659 (22.9%)	617 (14.4%)	775 (36.4%)	267 (32.2%)

* available only for metropolitan France; ** Index hospital stay = the first stay of the year in which the patient was hospitalized for MR.

Table S3. Association between sex, age, Charlson index comorbidity and type of management of patients hospitalized for mitral regurgitation, France, 2019.

a. Primary MR

	Primary MR							
	MVP				Rheumatic MR			
	**Crude OR**	**p value**	**Adjusted OR**	**p value**	**Crude OR**	**p value**	**Adjusted OR**	**p value**
**Surgical valve replacement**								
Sex (women vs men)	1.21 [1.01;1.44]	0.03	1.18 [0.99;1.41]	0.07	1.49 [1.08;2.05]	0.01	1.53 [1.11;2.11]	0.02
Age	1.01 [1.00;1.02]	<0.01	1.01 [1.00;1.01]	0.18	0.98 [0.97;0.99]	<0.01	0.98 [0.97;0.99]	<0.01
Charlson ≥2 vs <2	1.71 [1.43;2.04]	<0.01	1.64 [1.36;1.98]	<0.01	0.79 [0.58;1.06]	0.12	0.90 [0.66;1.22]	0.02
**Surgical mitral valve repair**								
Sex (women vs men)	0.54 [0.47;0.62]	<0.01	0.70 [0.60;0.82]	<0.01	0.42 [0.25;0.68]	<0.01	0.40 [0.24;0.67]	<0.01
Age	0.93 [0.92;0.94]	<0.01	0.94 [0.93;0.95]	<0.01	0.97 [0.96;0.99]	<0.01	0.98 [0.97;1.00]	0.01
Charlson ≥2 vs <2	0.26 [0.22;0.30]	<0.01	0.35 [0.30;0.42]	<0.01	0.26 [0.13;0.50]	<0.01	0.28 [0.14;0.55]	<0.01
**Percutaneous mitral valve repair**								
Sex (women vs men)	1.82 [1.50;2.19]	<0.01	0.94 [0.75;1.18]	0.61	0.31 [0.15;0.63]	<0.01	0.31 [0.15;0.64]	<0.01
Age	1.17 [1.16;1.19]	<0.01	1.17 [1.15;1.19]	<0.01	1.09 [1.05;1.14]	<0.01	1.09 [1.05;1.13]	<0.01
Charlson ≥2 vs <2	3.25 [2.68;3.94]	<0.01	1.82 [1.45;2.28]	<0.01	3.80 [1.78;8.11]	<0.01	2.50 [1.14;5.5]	0.02
**No mitral procedure mentioned**								
Sex (women vs men)	1.57 [1.27;1.94]	<0.01	1.45 [1.17;1.8]	<0.01	1.24 [0.87;1.77]	0.23	1.24 [0.87;1.78]	0.24
Age	1.02 [1.02;1.03]	<0.01	1.02 [1.01;1.02]	<0.01	1.03 [1.01;1.04]	<0.01	1.02 [1.01;1.03]	<0.01
Charlson ≥2 vs <2	1.91 [1.54;2.37]	<0.01	1.71 [1.36;2.15]	<0.01	1.59 [1.15;2.2]	<0.01	1.41 [1.01;1.96]	0.04

b. Secondary MR

	Ischemic MR				Secondary MR Cardiomyopathy MR				Other secondary MR			
	Crude OR	p value	Adjusted OR	p value	Crude OR	p value	Adjusted OR	p value	Crude OR	p value	Adjusted OR	p value
**Surgical valve replacement**												
Sex (women vs men)	1.05 [0.81;1.35]	0.73	1.17 [0.90;1.52]	0.23	1.48 [0.89;2.44]	0.13	1.59 [0.95;2.67]	0.10	1.10 [0.76;1.59]	0.61	1.28 [0.87;1.88]	0.23
Age	0.96 [0.95;0.97]	<0.01	0.96 [0.95;0.97]	<0.01	0.99 [0.97;1.00]	0.11	0.98 [0.96;1.00]	0.04	0.98 [0.96;0.99]	<0.01	0.97 [0.96;0.98]	<0.01
Charlson ≥2 vs <2	0.83 [0.65;1.06]	0.13	0.92 [0.72;1.19]	0.62	2.07 [1.25;3.44]	0.01	2.45 [1.43;4.19]	0.01	1.61 [1.11;2.34]	0.01	1.9 [1.29;2.80]	0.09
**Surgical mitral valve repair**												
Sex (women vs men)	0.51 [0.36;0.72]	<0.01	0.54 [0.38;0.77]	<0.01	0.44 [0.25;0.79]	<0.01	0.41 [0.22;0.76]	<0.01	0.50 [0.34;0.74]	<0.01	0.55 [0.37;0.83]	<0.01
Age	0.96 [0.94;0.97]	<0.01	0.96 [0.95;0.98]	<0.01	0.97 [0.95;0.99]	<0.01	0.98 [0.96;0.99]	0.02	0.96 [0.95;0.97]	<0.01	0.97 [0.95;0.98]	<0.01
Charlson ≥2 vs <2	0.38 [0.28;0.52]	<0.01	0.41 [0.30;0.57]	<0.01	0.30 [0.16;0.57]	<0.01	0.31 [0.16;0.60]	<0.01	0.29 [0.19;0.44]	<0.01	0.32 [0.2;0.49]	<0.01
**Percutaneous mitral valve repair**												
Sex (women vs men)	0.82 [0.61;1.11]	0.20	0.69 [0.51;0.94]	0.02	0.44 [0.21;0.89]	0.02	0.37 [0.18;0.78]	0.01	0.75 [0.46;1.24]	0.26	0.51 [0.3;0.88]	0.01
Age	1.06 [1.04;1.07]	<0.01	1.06 [1.04;1.08]	<0.01	1.06 [1.03;1.1]	<0.01	1.07 [1.03;1.11]	<0.01	1.09 [1.06;1.12]	<0.01	1.1 [1.06;1.13]	<0.01
Charlson ≥2 vs <2	1.77 [1.32;2.37]	<0.01	1.61 [1.19;2.17]	<0.01	2.07 [1.01;4.23]	0.05	1.75 [0.83;3.66]	0.14	1.73 [1.04;2.87]	0.03	1.57 [0.93;2.66]	0.09
**No mitral procedure mentioned**												
Sex (women vs men)	1.58 [1.24;2]	<0.01	1.46 [1.14;1.86]	<0.01	1.72 [1.09;2.69]	0.02	1.69 [1.07;2.65]	0.02	1.88 [1.32;2.67]	<0.01	1.63 [1.14;2.35]	0.01
Age	1.04 [1.02;1.05]	<0.01	1.03 [1.02;1.05]	<0.01	1.02 [1;1.03]	0.05	1.02 [1;1.03]	0.05	1.04 [1.03;1.06]	<0.01	1.04 [1.02;1.05]	<0.01
Charlson ≥2 vs <2	1.44 [1.13;1.82]	<0.01	1.35 [1.06;1.71]	0.02	0.84 [0.54;1.31]	0.44	0.77 [0.49;1.23]	0.27	1.31 [0.93;1.84]	0.13	1.17 [0.82;1.67]	0.39

Table S4. Association between sex, age, Charlson index comorbidity, type of management and mortality of patients hospitalized for mitral regurgitation, France, 2019.

	Crude OR	p-value	Adjusted OR	p-value
**MVP: Mortality during index stay**				
Sex (women vs. men)	2.19 [1.24;3.85]	0.01	1.65 [0.92;2.97]	0.09
Age	1.06 [1.03;1.09]	<0.01	1.05 [1.02;1.09]	<0.01
Charlson ≥2 vs. <2	2.8 [1.59;4.93]	<0.01	1.82 [1.01;3.3]	0.05
Surgical valve replacement vs. no act	0.88 [0.44;1.76]	0.72	1.24 [0.6;2.57]	0.57
Surgical valve repair vs. no act	0.14 [0.06;0.32]	<0.01	0.31 [0.13;0.78]	0.01
Percutaneous valve repair vs. no act	0.21 [0.07;0.65]	0.01	0.15 [0.05;0.47]	<0.01
**MVP: 30-day mortality**				
Sex (women vs. men)	1.95 [1.27;2.99]	<0.01	1.40 [0.89;2.20]	0.15
Age	1.08 [1.06;1.10]	<0.01	1.06 [1.03;1.09]	<0.01
Charlson ≥2 vs. <2	3.42 [2.22;5.26]	<0.01	2.07 [1.31;3.27]	<0.01
Surgical valve replacement vs. no act	0.41 [0.24;0.70]	<0.01	0.58 [0.33;1.02]	0.06
Surgical valve repair vs. no act	0.09 [0.05;0.16]	<0.01	0.20 [0.10;0.40]	<0.01
Percutaneous valve repair vs. no act	0.23 [0.12;0.46]	<0.01	0.16 [0.08;0.32]	<0.01
**MVP: One-year mortality**				

Sex (women vs. men)	1.54 [1.17;2.02]	<0.01	1.05 [0.78;1.41]	0.77
Age	1.09 [1.08;1.11]	<0.01	1.06 [1.04;1.08]	<0.01
Charlson ≥2 vs. <2	4.19 [3.19;5.52]	<0.01	2.3 [1.71;3.09]	<0.01
Surgical valve replacement vs. no act	0.39 [0.27;0.57]	<0.01	0.51 [0.35;0.76]	<0.01
Surgical valve repair vs. no act	0.07 [0.04;0.1]	<0.01	0.14 [0.09;0.23]	<0.01
Percutaneous valve repair vs. no act	0.55 [0.38;0.79]	<0.01	0.35 [0.24;0.51]	<0.01
**Rheumatic MR: One-year mortality**				
Sex (women vs. men)	0.90 [0.54;1.49]	0.68	1.02 [0.58;1.77]	0.95
Age	1.07 [1.05;1.1]	<0.01	1.06 [1.03;1.09]	<0.01
Charlson ≥2 vs. <2	4.81 [2.84;8.15]	<0.01	3.8 [2.18;6.63]	<0.01
Surgical valve replacement vs. no act	0.53 [0.31;0.89]	0.02	0.86 [0.48;1.53]	0.6
Surgical valve repair vs. no act	0.46 [0.17;1.22]	0.12	1.11 [0.38;3.25]	0.84
Percutaneous valve repair vs. no act	1.92 [0.8;4.64]	0.15	1.27 [0.5;3.22]	0.61
**Ischemic MR: One-year mortality**				
Sex (women vs. men)	1.29 [0.94;1.76]	0.12	1.15 [0.83;1.6]	0.4
Age	1.03 [1.01;1.04]	<0.01	1.03 [1.01;1.04]	0.01
Charlson ≥2 vs. <2	2.63 [1.87;3.7]	<0.01	2.57 [1.81;3.65]	<0.01
Surgical valve replacement vs. no act	0.73 [0.5;1.05]	0.09	0.91 [0.62;1.33]	0.62
Surgical valve repair vs. no act	0.43 [0.26;0.72]	<0.01	0.64 [0.37;1.1]	0.10
Percutaneous valve repair vs. no act	0.41 [0.25;0.66]	<0.01	0.37 [0.23;0.61]	<0.01
**Cardiomyopathy MR: One-year mortality**				
Sex (women vs. men)	0.87 [0.47;1.61]	0.65	0.76 [0.4;1.48]	0.42
Age	1.05 [1.02;1.08]	<0.01	1.04 [1.01;1.07]	0.02
Charlson ≥2 vs. <2	3.72 [1.85;7.46]	<0.01	2.93 [1.42;6.03]	<0.01
Surgical valve replacement vs. no act	0.98 [0.47;2.02]	0.95	0.98 [0.45;2.13]	0.96
Surgical valve repair vs. no act	0.17 [0.04;0.75]	0.02	0.25 [0.06;1.13]	0.07
Percutaneous valve repair vs. no act	1.59 [0.66;3.82]	0.30	1.10 [0.44;2.76]	0.85
**Other secondary MR: One-year mortality**				
Sex (women vs. men)	1.79 [1.06;3.02]	0.03	1.53 [0.88;2.64]	0.13
Age	1.03 [1.01;1.05]	0.01	1.02 [0.99;1.04]	0.17
Charlson ≥2 vs. <2	2.81 [1.65;4.76]	<0.01	2.33 [1.35;4.01]	<0.01
Surgical valve replacement vs. no act	0.8 [0.44;1.44]	0.45	0.91 [0.48;1.72]	0.77
Surgical valve repair vs. no act	0.18 [0.07;0.48]	<0.01	0.29 [0.10;0.78]	0.01
Percutaneous valve repair vs. no act	0.96 [0.45;2.01]	0.90	0.91 [0.43;1.96]	0.81

Table S5. Characteristics of patients hospitalized for mitral valve prolapse according to heart failure, France, 2019.

	**Mitral Valve Prolapse**		
	**History of Heart Failure**	**No History of Heart Failure**	***p*-Value**
**Total number, n**	1098	2462	
**Demographic characteristics**			
Age (years), mean (SD)	74.3 (11.8)	65.0 (13.3)	<0.01
Women, n (%)	440 (40.1%)	782 (31.8%)	<0.01
**Medical characteristics**			
Charlson comorbidity index mean (SD)	3.34 (1.99)	1.20 (1.76)	<0.01
Length of index stay mean (SD)	10.6 (12.3)	8.3 (9.7)	<0.01
**Management of mitral regurgitation in the year following the index hospital stay, n (%)**			
Surgical valve replacement	268 (24.4%)	409 (16.6%)	<0.01
Surgical valve plasty	355 (32.3%)	1,606 (65.2%)	

Percutaneous mitral valve repair	319 (29.1%)	203 (8.2%)	
No mitral procedure mentioned	156 (14.2%)	244 (9.9%)	
**Readmission in the year following**			
After index stay, all causes, n (%)	677 (61.7%)	1,457 (59.2%)	0.16
After index stay, for RM, n (%)	320 (29.1%)	1,000 (40.6%)	<0.01
After surgical or percutaneous act, all causes, n (%)	409 (43.4%)	653 (29.4%)	<0.01
After surgical or percutaneous act, for RM, n (%)	29 (3.1%)	44 (2.0%)	0.06
After surgical or percutaneous act, MACCE, n (%)	184 (19.5%)	189 (8.5%)	<0.01
**All causes mortality, n (%)**			
**After index stay**			
During the index hospital stay	25 (2.3%)	24 (1.0%)	<0.01
At 30 days	46 (4.2%)	40 (1.6%)	<0.01
At 1 year	131 (11.9%)	95 (3.9%)	<0.01
**After surgical or percutaneous act**			
At 30 days	34 (3.6%)	32 (1.4%)	<0.01
At 1 year	84 (8.9%)	68 (3.1%)	<0.01

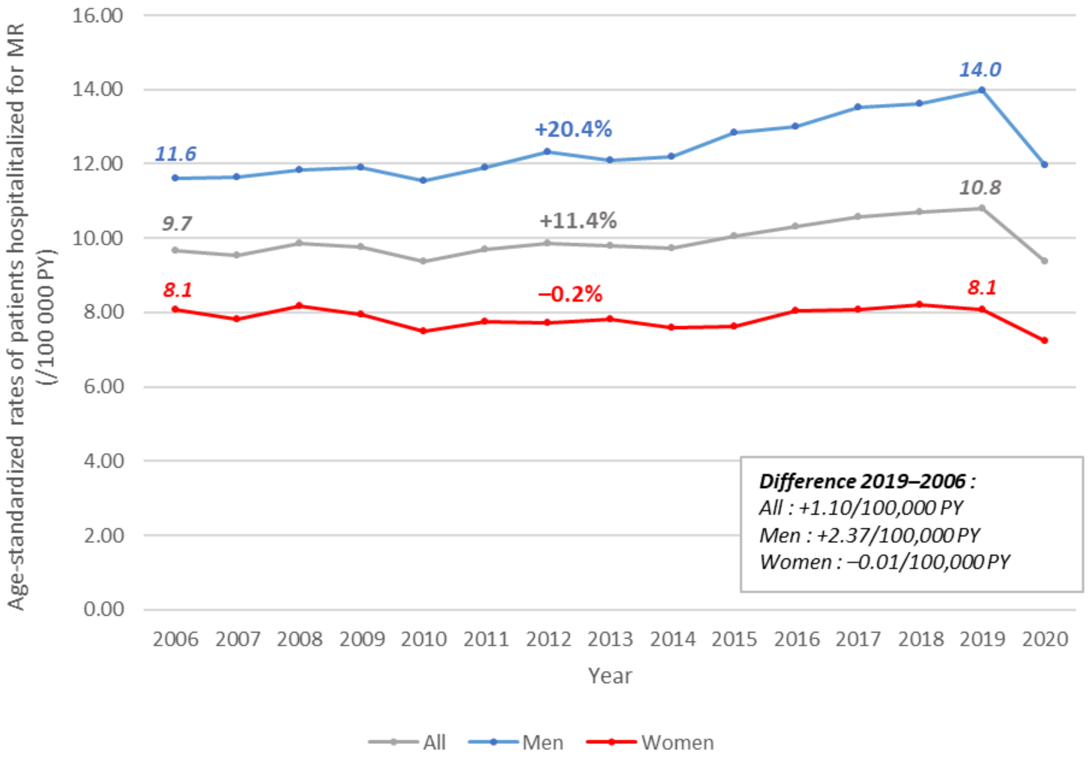

Figure S1. Trends in age-standardized rates of patients hospitalized for mitral regurgitation (all) in person-years, according to sex, France, 2006–2020. *The percentage above the curve is the relative difference in rates between 2019 and 2006. PY = person-years.*

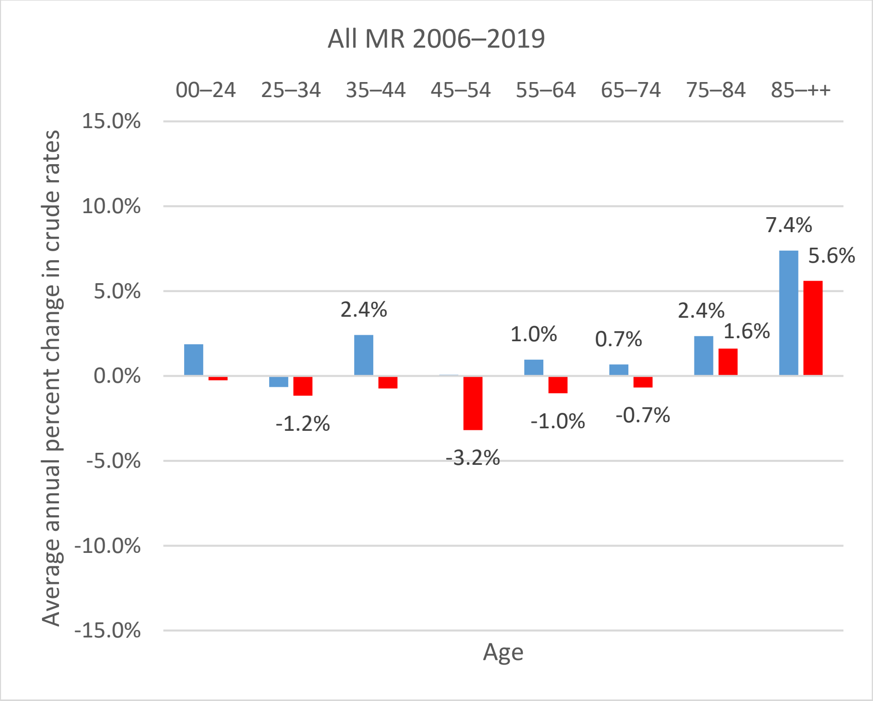

Figure S2. Average annual percent change in crude rates of patients hospitalized for mitral regurgitation (all), according to sex, France, 2006–2019. *Only values significantly different from 0% are presented (alpha risk = 5%)*

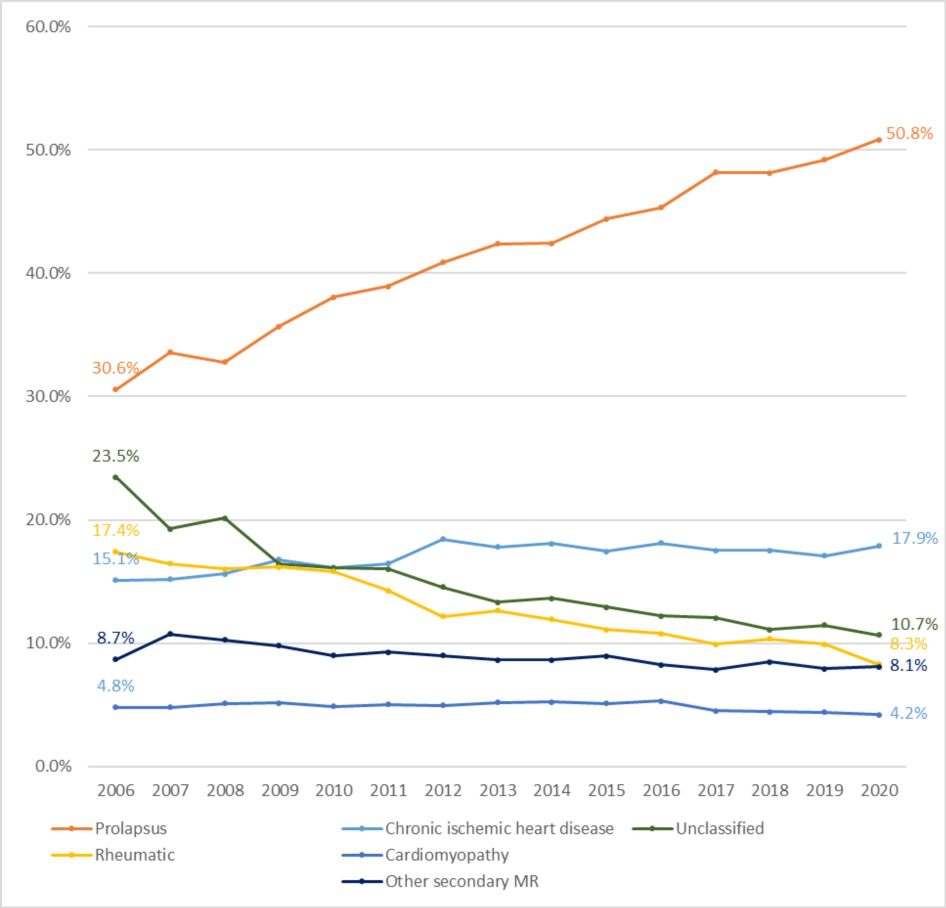

Figure S3. Trends in the distribution of hospitalized mitral regurgitation etiologies, France, 2006–2020.

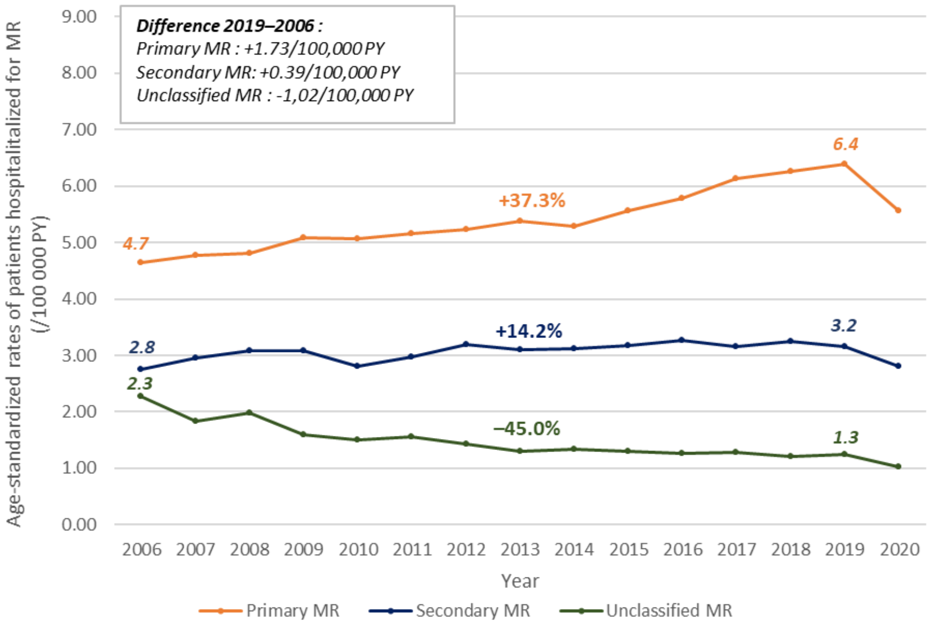

Figure S4. Trends in age-standardized rates of patients hospitalized for mitral regurgitation in person-years, according to type of MR and sex, France, 2006–2020. *The percentage above the curve is the relative difference in rates between 2019 and 2006. PY = person-years.*

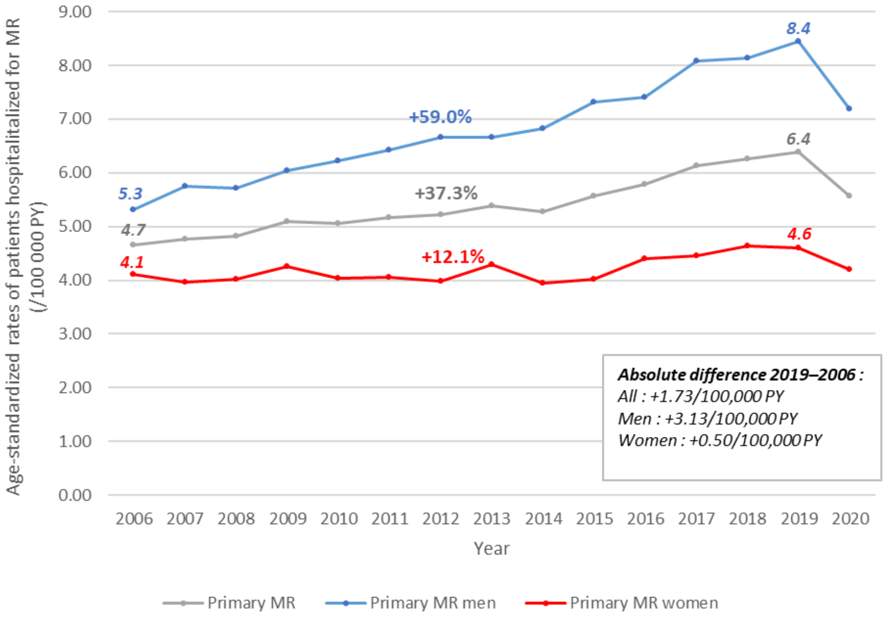

Figure S5. Trends in age-standardized rates of patients hospitalized for primary mitral regurgitation in person-years, according to etiology and sex, France, 2006–2020. *The percentage above the curve is the relative difference in rates between 2019 and 2006. PY = person-years.*

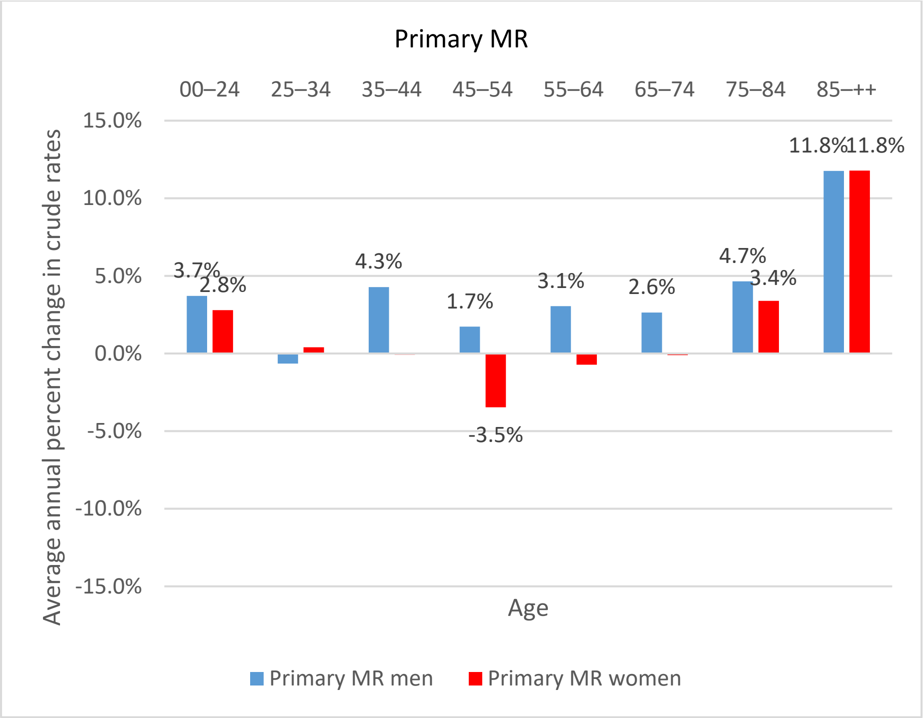

Figure S6. Average annual percent change in crude rates of patients hospitalized for secondary mitral regurgitation, according to sex, France, 2006–2019. *Only values significantly different from 0% are presented (alpha risk = 5%).*

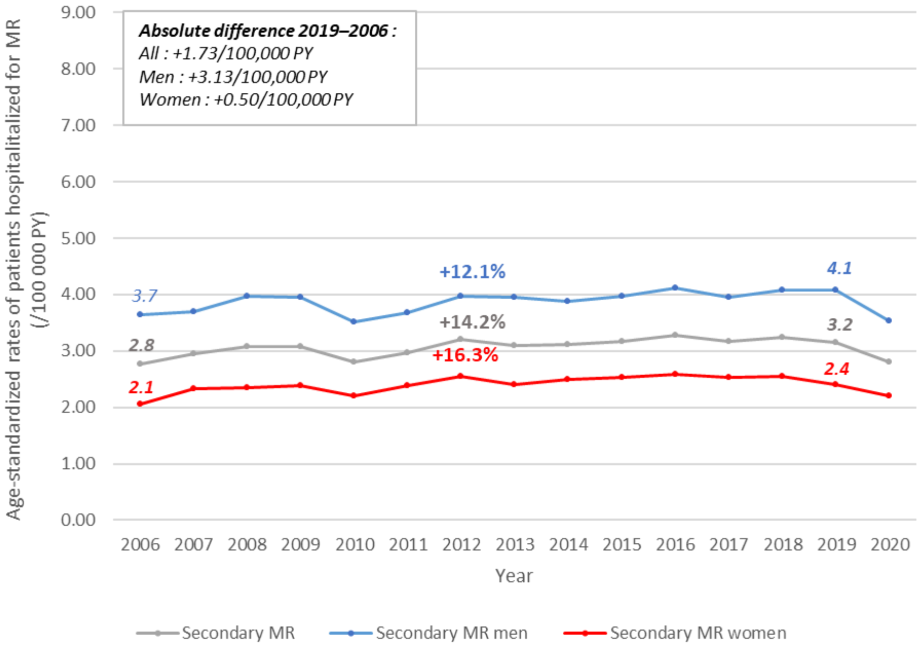

Figure S7. Trends in age-standardized rates of patients hospitalized for secondary mitral regurgitation in person-years, according to etiology and sex, France, 2006–2020. *The percentage above the curve is the relative difference in rates between 2019 and 2006. PY = person-years.*

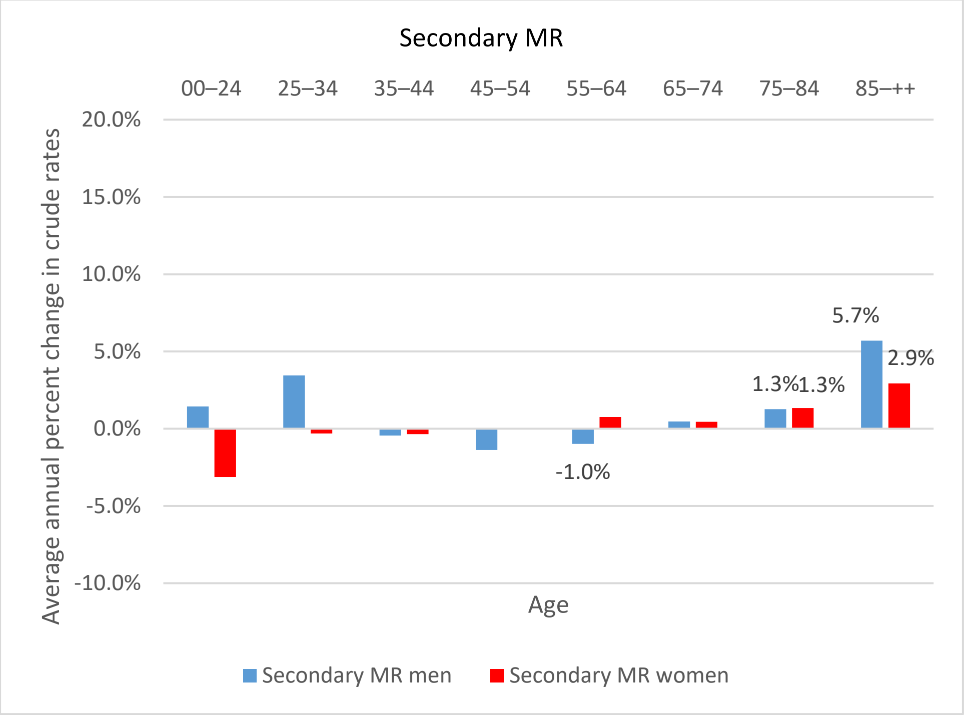

Figure S8. Average annual percent change in crude rates of patients hospitalized for secondary mitral regurgitation, according to sex, France, 2006–2019. *Only values significantly different from 0% are presented (alpha risk = 5%).*
